# Transformer-Based Approach to Melanoma Detection

**DOI:** 10.3390/s23125677

**Published:** 2023-06-17

**Authors:** Giansalvo Cirrincione, Sergio Cannata, Giovanni Cicceri, Francesco Prinzi, Tiziana Currieri, Marta Lovino, Carmelo Militello, Eros Pasero, Salvatore Vitabile

**Affiliations:** 1 Département Electronique-Electrotechnique-Automatique (EEA), University of Picardie Jules Verne, 80000 Amiens, France; giansalvo.cirrincione@u-picardie.fr; 2 Department of Electronics and Telecommunications, Politecnico di Torino, 10129 Turin, Italy; eros.pasero@polito.it; 3 Department of Biomedicine, Neuroscience and Advanced Diagnostics (BiND), University of Palermo, 90127 Palermo, Italy; giovanni.cicceri@unipa.it (G.C.); francesco.prinzi@unipa.it (F.P.); tiziana.currieri@unipa.it (T.C.); salvatore.vitabile@unipa.it (S.V.); 4 Department of Engineering Enzo Ferrari, University of Modena and Reggio Emilia, 41125 Modena, Italy; marta.lovino@unimore.it; 5 Institute for High-Performance Computing and Networking (ICAR-CNR), Italian National Research Council, 90146 Palermo, Italy; carmelo.militello@cnr.it

**Keywords:** skin cancer, melanoma detection, vision transformers, artificial intelligence, decision-making support

## Abstract

Melanoma is a malignant cancer type which develops when DNA damage occurs (mainly due to environmental factors such as ultraviolet rays). Often, melanoma results in intense and aggressive cell growth that, if not caught in time, can bring one toward death. Thus, early identification at the initial stage is fundamental to stopping the spread of cancer. In this paper, a ViT-based architecture able to classify melanoma versus non-cancerous lesions is presented. The proposed predictive model is trained and tested on public skin cancer data from the ISIC challenge, and the obtained results are highly promising. Different classifier configurations are considered and analyzed in order to find the most discriminating one. The best one reached an accuracy of 0.948, sensitivity of 0.928, specificity of 0.967, and AUROC of 0.948.

## 1. Introduction

According to the World Health Organization (WHO), skin cancer is considered one of the most common types of cancer worldwide [1]. Over one million skin cancer cases are diagnosed each year, estimated to account for one third of all cancer diagnoses [2]. Exposure to sun ultraviolet (UV) radiation represents the primary cause of skin cancer. Hence, the WHO recommends avoiding prolonged sun exposure to individuals with fair skin and regular self-examinations for any suspicious lesions on the skin.

Melanoma is a skin cancer that develops from the pigment-producing cells known as melanocytes [3]. The incidence of melanoma has significantly increased in the past 30 years [4], making up only 5 percent of all cases but having a mortality rate of over 75 percent [5,6].

Melanoma skin cancer requires early diagnosis and treatment, aspects vital to improving outcomes in affected individuals and preventing it from rapidly spreading to other areas of the body. To this aim, detecting and classifying it could represent a useful tool supporting decision making in the diagnosis phase and the whole treatment process [7,8].

Computer-aided diagnosis (CAD) technologies are utilized to help with the detection of melanoma skin cancer. These systems integrate artificial intelligence (AI), with machine learning (ML) and deep learning (DL) models used to analyze skin images, detect abnormal tissue patterns, and classify them into cancerous or non-cancerous areas [9,10]. Among the DL methods, convolutional neural networks (CNNs) have shown promising results in solving medical imaging tasks [11,12,13]. However, CNNs have limitations in understanding long-range spatial relationships in images [14].

Indeed, CNNs investigate images by applying filters that are able to capture specific features, such as edges or shapes. However, such filters must often deal with the downside of having a limited receptive field. This condition prevents the model from effectively connecting information associated with features located in distant areas of the image with what has been extracted locally. Although specific methods (e.g., filter stacking and factorization) have been developed to deal with this issue, CNNs still exhibit a lack of comprehension of the global picture context. To further complicate matters, the idea of modifying CNN filters to accommodate for wider receptive fields would lead to a number of parameters that would be unbearably large from a computational cost perspective. Aside from that, tackling the problem of grasping long-range spatial relationships among different parts of an image by leveraging recurrent neural networks (RNNs) has been explored in the literature.

Despite their aforementioned challenges, several studies have explored the use of RNNs for image classification. The authors of [15] proposed a recurrent convolutional neural network (RCNN) architecture that combines the strengths of CNNs and RNNs for image classification tasks. The proposed RCNN model integrates recurrent connections to capture the long-range dependencies within images, thereby augmenting the network’s capacity to comprehend the global context and spatial relationships. The authors of [16] proposed a method to enhance the performance of RNNs by using two parallel recurrent layers that operate independently, where the final output of each recurrent layer is computed as the mean of the modulus of their respective outputs. RNNs still display a higher rate of success in applications other than vision and image analysis, such as time series forecasting and speech recognition. This can be supported by observing that RNNs can easily experience vanishing gradient issues. For this reason, as much as an RNN is able to retain the memory of past inputs, working on long sequences can still cause the model to forget relevant information. In the vision domain, this implies that a recurrent network might face major difficulties when attempting to capture the connections among image areas located far from each other.

Long short-term memory (LSTM) models were born to process sequences and pay attention to their sequential inputs, even at long distances. However, these models are sequential and process one input at a time. Therefore, although they have memory cells designed to keep track of the sequential information, updating the weights progressively considers the contribution of long-range dependencies less and less. By contrast, transformer architectures use the self-attention mechanism to propagate information over time, and each region of the sequence pays attention to all other regions. In this way, all the dependencies—even those at a very long range—are modeled without loss of information. Furthermore, transformers are parallelizable, as they can process multiple inputs simultaneously. Given these characteristics, the advantages of using transformers led us to choose this architecture instead of LSTM or the RNN.

The proposed method—based on Vision Transformers (ViTs) [17]—addressed the limitations of conventional RNNs in terms of gradient flow by surpassing classical LSTM and the identity RNN (IRNN) in terms of performance on several benchmark image datasets. Indeed, the ViT feature extraction method is based on linear embedding of the patches in which the input image is divided. This implies that such a process still occurs locally, as for CNNs. However, additional encoding is further applied on patches to account for their positional information. Moreover, the patches’ linear transformations are learned by the model through backpropagation. Such a procedure further enhances the influence of spatial information among the patches, regardless of their location in the picture. For these reasons, the Vision Transformer has been identified as a powerful tool to deal with challenging image analysis applications.

Various approaches have been implemented using ViTs to classify images. The authors of [18] presented a comprehensive investigation into the robustness of ViTs for image classification. The authors evaluated the models’ ability to withstand both input perturbations and model perturbations. The results highlighted that ViTs, when adequately pretrained with large data, exhibit similar or superior robustness when compared with ResNet models across various perturbations. Additionally, this study revealed that transformers demonstrate resilience when specific layers are removed, indicating their flexibility. Although activations from later layers exhibit strong correlations, it is evident that each layer contributes significantly to the classification process. The authors of [19] introduced a comprehensive framework (called the Classification Transformer (C-Tran)) for multi-label image classification that leverages transformers to capture the intricate relationships between the visual features and labels. The proposed approach involves training a transformer encoder using masked labels and the visual features from a CNN to predict a set of target labels by achieving state-of-the-art performance on challenging datasets such as COCO and Visual Genome. Despite existing research exploring the use of ViTs in the field of melanoma skin cancer detection and classification [20,21], the literature lacks approaches specifically focused on multiclass classification. To fill this research gap, in this paper, a ViT-based architecture for the recognition of melanoma versus non-cancerous lesions is presented.

Specifically, the main contributions of the proposed work are as follows:

- The proposed model is trained and tested on public skin cancer data from the ISIC challenge, making a fair comparison with competitors with similar approaches to dealing with the same clinical issue possible;
- The performance of the presented ViT-based architecture for skin lesion image classification exceeds that of the state of the art by modeling long-range spatial relationships;
- Extensive tuning studies are performed to investigate the effect of individual elements of the ViT model.

The remainder of this paper is organized as follows. Section 2 provides the characteristics of the dermatoscopic melanoma image dataset used and processed in this study. Section 3 describes the implemented methodology to set up the DL predictive model, detailing each step of the processing pipeline. Section 4 illustrates the obtained experimental results concerning each specific step and the performance of the built-up model, both in the training and validation phase and in the test phase. Section 5 delves into the obtained results, offering comprehensive analysis from both the technical and clinical perspectives. Finally, Section 6 provides a concise summary of the study’s key findings and draws meaningful conclusions.

## 2. Dataset

The dataset considered for this study is based on a collection of dermoscopic images of skin lesions obtained from the ISIC Archive (ISIC Archive website https://challenge.isic-archive.com/data/#2017, accessed on 1 June 2023). Specifically, the dataset served as the basis for the ISIC 2017 Challenge (Skin Lesion Analysis toward Melanoma Detection) [22], where researchers and clinicians worldwide competed to develop innovative algorithms and tools for the automated diagnosis of skin lesions, including melanoma. Metadata, including the patient’s age, sex, and lesion location, accompany each image. The images were labeled by expert dermatologists who identified the presence or absence of melanoma based on histopathology examination or clinical examination. This procedure ensured the accuracy and reliability of the labels. The availability of such a comprehensive and annotated dataset has facilitated research efforts in this field, leading to the development of more accurate and efficient diagnostic tools for melanoma and other skin cancers. The ISIC 2017 Challenge consisted of three tasks: lesion segmentation, dermoscopic feature detection, and lesion classification. In this article, we focused on the lesion classification task, aiming at the classification of skin lesion images into three classes: malignant melanoma (MM), nevocellular nevus (NCN), and seborrheic keratosis (SK). Melanoma is defined as a malignant skin tumor derived from melanocytes (melanocytic). Seborrheic keratosis (SK) is defined as a benign skin tumor derived from keratinocytes (non-melanocytic). A nevus is defined as a benign skin tumor derived from melanocytes (melanocytic).

The training dataset contains 2000 color dermoscopic skin images in JPEG format, comprising 374 images from melanoma cases, 254 from seborrheic keratosis cases, and the remainder being benign nevi (1372). The validation and test sets were evaluated in new databases with 150 images (30 for MM, 42 for SK, and 78 for NCN) and 600 images (117 for MM, 90 for SK, and 393 for NCN), respectively [22]. The images were of many sizes (from 1022 × 767 to 6748 × 4499 pixels) and photographed with different angles, artifacts, and lighting conditions. The images were acquired from various dermatology clinics and hospitals across different continents, ensuring diversity in the dataset.

**Figure 1:**
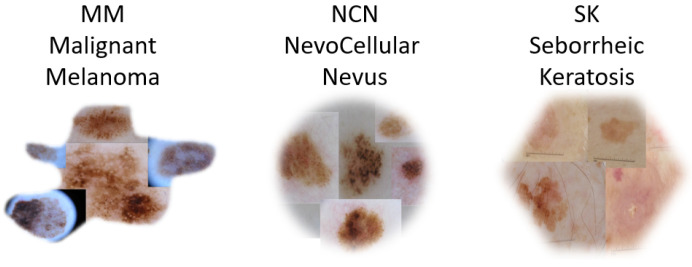
Some examples belonging to the used dataset, with the three classes highlighted.

Figure 1 represents examples from the three classes: MM, NCN, and SK. Inhomogeneous dark spots with irregular shapes usually characterized the MM class, and similar features were shared with the NCN and SK classes, making the classification task particularly challenging.

## 3. Methodology

The proposed methodology is based on ViTs, used to encode images which are able to model long-range spatial relationships among areas in the dermatoscopic image, with respect to traditional deep-learning architectures. Before proceeding with the model’s set-up, the images were properly processed (i.e., resized and cropped).

**Figure 2:**
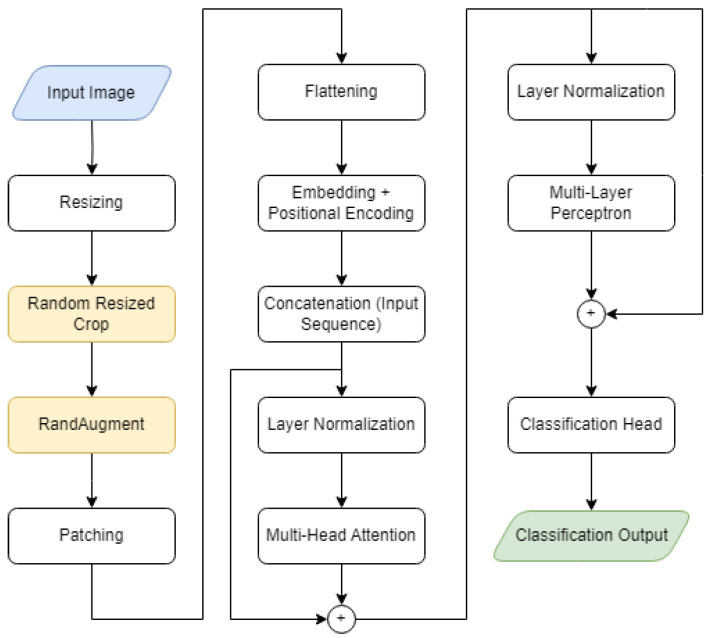
The implemented end-to-end workflow.

Finally, the model exploiting the ViT and the MLP head was trained and validated. The end-to-end workflow is represented in Figure 2 and described in the next subsection.

### 3.1. The End-to-End Workflow

The dataset splits were unmodified with respect to the original database, as described in the previous sections. The images went through the following processing procedure:

- **Input image**: The input image was fed to the model at its original size. A blue-colored block represented the workflow’s starting point.
- **Resizing**: The images were resized to 384 × 384 in order to be properly processed by the model.
- **Random resized cropping**: The training images were randomly resized and cropped as a part of the data augmentation procedure. This block was marked with yellow to indicate that it only operated on the training images.
- **RandAugment**: The RandAugment library [23] was applied to the training set for data augmentation purposes. This block, as with the previous one, was marked in yellow, with the exact same meaning.
- **Patching**: The original image was divided into equally sized, non-overlapping 32 × 32 square patches.
- **Flattening**: Each patch was vectorized (flattening) in order to have an embedding for the corresponding token in the input sequence.
- **Embedding + positional encoding**: The patches were encoded to account for their positional information.
- **Concatenation (input sequence)**: The positional encoded vector was added to the corresponding token.
- **Layer normalization**: The resulting vector was *layer normalized* and fed to the transformer encoder.
- **Multi-head attention**: The multi-head attention mechanism came into play, reproducing the behavior of multiple “experts” analyzing different areas of the picture, exchanging information, and interacting to come up with a coherent interpretation of the image.
- **Layer normalization**: The output of the multi-head attention blocks was added to the embedded input by means of a skip connection, and it was later normalized once more.
- **Multilayer perceptron**: The resulting output was fed to an MLP consisting of two fully connected layers with a GELU activation function [24].
- **Classification head**: The MLP output was added to the multi-head attention output through another skip connection. The classification head is a fully connected layer that will produce the model output.
- **Classification output**: The final outcome was a value that would be used to determine which class the input image belonged to. This block, highlighted in green, marks the end of the workflow.

**Figure 3:**
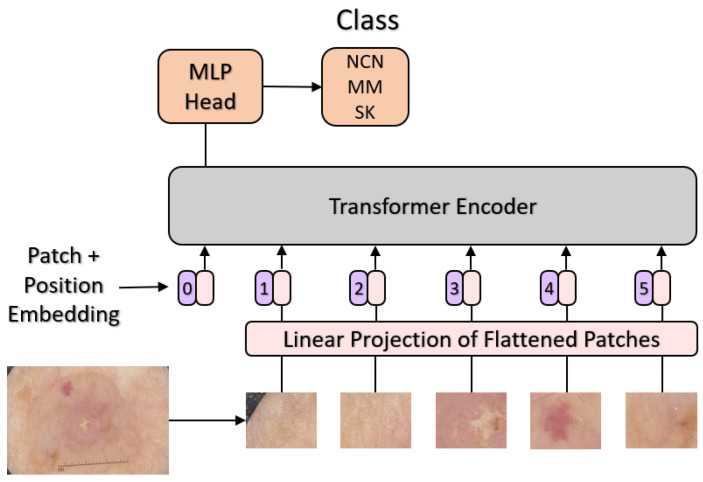
Overall scheme of the proposed ViT-based architecture for melanoma classification.

The next section will explore the dynamics of *multi-head attention* (see Figure 3).

To this aim, mentioning that ViTs are an adaptation to the computer vision domain of the original Transformer [25] can help to grasp the underlying concepts of the architecture. In fact, the very first transformers were and still are applied to Natural Language Processing, a field in which knowledge about the *position* of a word within a sentence, together with the capability to understand the *context*, is the key to a good interpretation.

#### Multi-Head Attention

To achieve the goal introduced above, each input was associated with three main quantities, namely the *key*, the *query*, and *value*. Those quantities were obtained by multiplying the input by a set of weights that needed to be initialized. The next step was the multiplication of each query by each key through a dot product scaled by the square root of the key vector dimension. The result was later fed to a softmax function and multiplied by the value vector. The final outcome is called the *attention score*, and each block of layers performing the described sequence of operations is called a *head*. The whole sequence can be executed by several heads at a time (hence the name *multi-head attention*). In a sense, this process resembles a team of specialists working on the same picture, with each one of them focusing on a specific subsection of the image, drawing conclusions, and sharing knowledge with the others.

### 3.2. Experimental Set-Up

In order to determine the optimal configuration, the experimental phase was subdivided into two main steps: (1) a search for the best learning rate and (2) an ablation study, with the dual purpose of investigating the effect of the network depth on performance and determining the proper trade-off between model complexity and efficiency. Specifically, to determine the best learning rate, we conducted a systematic search by training the model with different learning rate values. We started with a relatively large learning rate ($1.5\times 10^{-5}$) and gradually reduced it to $0.5\times 10^{-6}$, monitoring the model’s performance at each iteration. We evaluated the model’s performance metrics based on accuracy on a validation set to identify the learning rate that resulted in the best performance. The ablation study served two main purposes: (1) examining the impact of the network depth on performance to understand how it influenced the model’s effectiveness and (2) striking a balance between model complexity and efficiency. Through these two steps in the experimental phase, our objective was to refine the model and identify the optimal configuration that maximized performance while taking into account computational efficiency.

#### Evaluation Metrics

The performance of the proposed approach was evaluated using conventional metrics such as accuracy, sensitivity, and specificity, defined in Equations (1)–(3), respectively. Moreover, the area under the receiver operating characteristic curve (AUROC) was calculated [26]:

(1) $$Accuracy=\frac{TN+TP}{TN+TP+FN+FP}$$

(2) $$Sensitivity=\frac{TP}{TP+FN}$$

(3) $$Specificity=\frac{TN}{TN+FP}$$

where *TP*, *TN*, *FP*, and *FN* represent the number of true positives, true negatives, false positives, and false negatives, respectively.

## 4. Experimental Results

The experiments were conducted in the Google Colab environment while deploying the models available on Hugging Face. The best selected model was ViT-Large (307 million parameters), with an input resolution of 384 × 384 and a patch size of 32 × 32.

The proposed model, which was pretrained on ImageNet [27], was fine-tuned on the ISIC 2017 dataset for 40 epochs using the hyperparameters listed in Table 1. The ViT-based model was trained using several configurations, which are listed in Table 2. In particular, the performance was evaluated by varying the learning rate of the Adam optimizer and changing the number of hidden layers. The scheduler, which was used for the training phase, applied a linear learning rate variation with a warm-up. When the set point for the learning rate (maximum value to be reached) was chosen, the scheduler started from a lower value until the set point was reached and decreased with a gentler slope in the last few epochs. In particular, having set the number of layers to 24, different set points were selected (i.e., $0.5\times 10^{-6}$, $0.5\times 10^{-5}$, $1.0\times 10^{-5}$, and $1.5\times 10^{-5}$). Such a scheduling method guaranteed that the experiments explored the influence of the learning rate over a sufficient dynamic range. In general, the effect of high learning rate values was reflected in a learning curve trend that did not decrease effectively as a consequence of the model not learning proper classification. On the other hand, low learning rate values can lead the system to learn rather efficiently (aside from the risk of getting stuck in local minima). However, this usually requires a large number of epochs to reach convergence. Considering this, since the system obtained the same accuracy value (94.83%) with the values $0.5\times 10^{-5}$, $1.0\times 10^{-5}$, and $1.5\times 10^{-5}$, we decided to choose the configuration with an intermediate learning rate. The proposed intermediate learning rate value requires a reasonable number of epochs for training.

**Figure 4:**
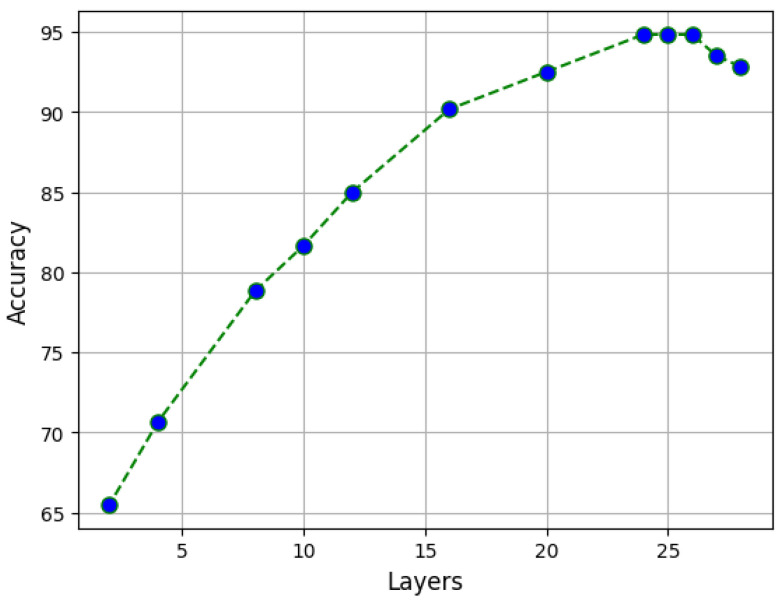
Variation in accuracy as a function of the number of layers.

A large difference in performance, on the other hand, was found as the number of layers varied. Figure 4 shows how the accuracy varied as the number of layers changed. In particular, different values in the range of 2–28 were tried. As shown in Figure 4, the accuracy had an increasing trend as the number of layers increased (as expected), and the increase was greater as the first few layers increased, whereas the growth subsequently became slower. In the range of 24–26, the accuracy presented its maximum (where the curve has a plateau), and the accuracy stayed constant at 94.83% while for higher values, the accuracy began to decrease. Indeed, this was a consequence of overfitting because we were increasing the model complexity. Considering these findings, and following *Occam’s razor*, a value of 24 was chosen for the number of hidden layers (which is also the default value for the ViT-Large architecture), minimizing the architecture’s complexity and optimizing the classification performance.

**Figure 5:**
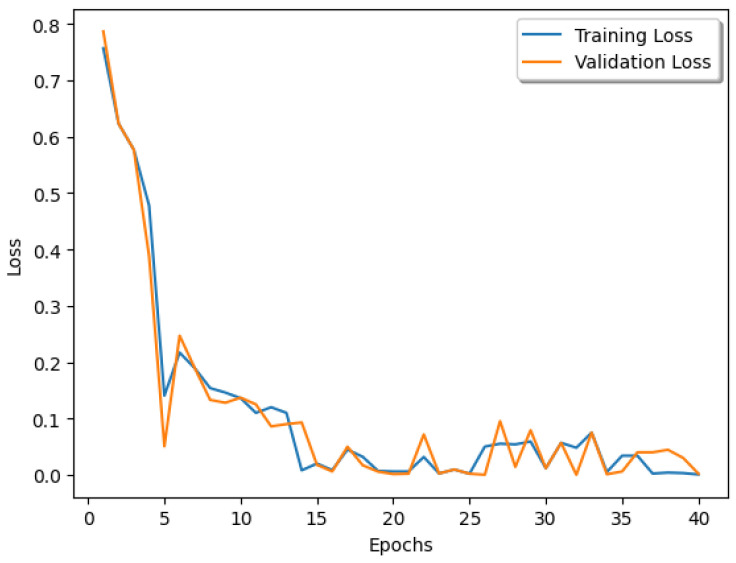
Training and validation loss of configuration 2 (best model).

**Figure 6:**
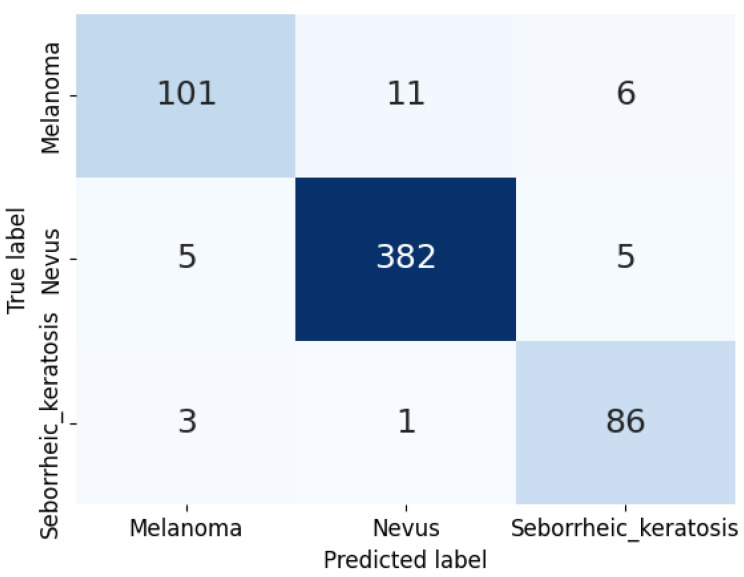
Confusion matrix for configuration 2 (best model).

Regarding the best configuration, the model reached a final test accuracy of 0.948. Figure 5 shows the training and validation losses. It is possible to observe a drop in both curves at epoch 5, which was likely due to the warm-up ratio of the learning rate scheduler. Moreover, the curves’ peaks that can be seen on the plot might be associated with the selected batch size. The test confusion matrix for configuration 2 is shown in Figure 6.

## 5. Discussion

In the literature, various methods have been proposed to improve the decision-making process in the diagnosis of skin lesions, particularly in cases of melanoma. The authors of [28] proposed a comprehensive comparative study of U-Net and attention-based skin lesion image segmentation methods. The results indicated that the hybrid TransUNet outperformed the other benchmarking methods in segmentation tasks, achieving an accuracy of 0.921 and a Dice coefficient of 0.898. The authors of [29] proposed the use of fully Transformer networks (FTNs) for skin lesion analysis, a hierarchical transformer that uses a spatial pyramid transformer (SPT) to capture long-range contextual information from skin lesion images. Their conducted experiments on the public International Skin Imaging Collaboration (ISIC) skin lesion segmentation and classification datasets demonstrated the effectiveness and efficiency of FTNs. In [30], the authors presented a novel deep learning-based framework named Attention Deeplabv3+ for skin lesion segmentation. Their proposed method uses attention mechanisms in two stages to capture the relationships between channels and to emphasize the relevant field of view more. Their experimental results on public skin cancer datasets showed high state-of-the-art performance. The authors of [31] proposed a novel self-attention-based network for diagnosing melanocytic lesions from digital whole slide images. The method outperformed other state-of-the-art methods and achieved results comparable to 187 practicing U.S. pathologists. In [32], the authors proposed a framework that employs methods for data augmentation and a Medical Vision Transformer-based classification model for classifying skin cancer. The results achieved on the HAM10000 datasets showed that the proposed model outperformed the state-of-the-art techniques for skin cancer classification, proving that early detection can increase survival rates by up to 70%, leading to improved outcomes for patients with this deadly disease. However, the potential of ViTs has not yet been exploited to its fullest in the classification of melanoma skin cancers.

Melanoma detection is a critical task in improving cancer diagnoses and prognoses. To this aim, this work proposed a ViT-based architecture for efficient MM recognition compared with the NCN and SK classes.

The proposed model (training and testing procedures) was built up using publicly available skin cancer data from the ISIC challenge. This choice enabled a fair comparison with other competitors who have employed similar methods to address the same clinical issue by using the same dataset. The performance in classifying skin lesion images of the introduced architecture based on ViTs outperformed the current state-of-the-art models. This improvement can be attributed to the model’s ability to capture and model long-range spatial relationships within the images effectively.

Table 3 shows the performance of our proposed ViT-based approach and a comparison with the other state-of-the-art solutions that used the ISIC 2017 dataset. It should be noted that in the specific application scenario approached by our study, there was no possibility of changing (increasing) the number of classes (i.e., the MM, NCN, and SK classes). Indeed, the number of classes was established in the ISIC 2017 skin lesion classification challenge to which the dataset refers. The optimal configuration is able to accurately identify images from all three categories, showing remarkable classification capabilities on the given dataset. The accuracy of 0.948, sensitivity of 0.928, and specificity of 0.967 were significantly higher compared with the other models. Only the AUROC results were a bit lower compared with the other models. Reducing the number of layers led to a decrease in accuracy. However, it is interesting to observe that employing a ViT-based architecture with 20 layers instead of 24 still yielded a final accuracy above the state of the art.

An in-depth analysis was given in order to assess the influence of the hyperparameters and to consequently select the best model configuration in terms of batch size, learning rate, and number of layers. First of all, the influence of the learning rate during the training phase was evaluated. After selecting the best learning rate value, further investigations were conducted to explore model performance and the number of layers within the architecture. This process was motivated by the necessity to balance efficacy and complexity. Indeed, despite the apparent direct proportionality between performance and network depth, the availability of a tool that can guarantee comparable results when deployed on devices with limited computational resources (e.g., a mobile phone, tablet, or smartwatch, which are increasingly used in e-Health and m-Health) is paramount. Furthermore, extensive ablation studies were conducted to explore the impact of individual components of the ViT model, which highlighted their respective contributions to the overall performance.

The proposed architectural solution represents a trade-off between computational cost and classification performance. The study performed in the tuning phase of the hyperparameters allowed obtaining a predictive model with very high sensitivity and specificity, corresponding to very low amounts of *falses* (positive as well as negative). This is due to the self-attention mechanism which, by taking into account the correspondences between patches, can better understand the image’s content. The transformer generally works in overfitting. Our ablation analysis also confirmed this, which shows that not all layers are needed. Considering that the last layers on the transformer represent the most abstract relations in the image, it can be deduced that the keys of the better classification require only the low-level features of the image. However, we cannot forget the high computational cost of the transformer, which implies that the proposed classification is offline.

Despite the better results of the proposed approach at convergence, the dynamics of the training (see Figure 5) is an issue because of the presence of high peaks. This phenomenon was probably due to the small size and the internal covariate shift of the mini-batches. Future works will deal with regularization techniques to overcome this problem, such as data augmentation using generative networks (e.g., GANs and diffusers).

## 6. Conclusions

Melanoma detection is crucial to improving cancer diagnoses and prognoses. This work presented a ViT-based model to efficiently recognize malignant melanoma, nevocellular nevus, and seborrheic keratosis cases. The model was evaluated on a known dataset from the literature called the ISIC challenge to ensure comparison with the state of the art. The results show how the proposed architecture outperformed the state-of-the-art models mainly based on convolutional networks. Ablation studies conducted on the presented model with its hyperparameters highlighted the robustness of the model for classification purposes. Future plans could include the use of attention maps to provide explainability to the model and understand which regions of the images are relevant for classification purposes.

## Figures and Tables

**Table 1:** Model hyperparameters.

Hyperparameter	Value
Number of layers	24
Number of heads	16
Hidden size	1024
Optimizer	Adam with $\beta_{1}$ = 0.99, $\beta_{2}$ = 0.999, ϵ= $1\times 10^{-8}$
Learning rate	$1.0\times 10^{-5}$
Learning rate scheduler	linear with a warm-up ratio of 0.1
Batch size	16
Gradient accumulation steps	4

**Table 2:** Tested configurations.

Configuration	Batch Size	Learning Rate	Layers	Accuracy
#1	16	$1.5\times 10^{-5}$	24	0.948
**#2 (Best Model)**	**16**	${1.0\times 10}^{-5}$	**24**	**0.948**
#3	16	$0.5\times 10^{-5}$	24	0.948
#4	16	$0.5\times 10^{-6}$	24	0.945
#5	16	$1.0\times 10^{-5}$	20	0.925
#6	16	$1.0\times 10^{-5}$	16	0.902
#7	16	$1.0\times 10^{-5}$	12	0.850
#8	16	$1.0\times 10^{-5}$	10	0.817
#9	16	$1.0\times 10^{-5}$	8	0.788
#10	16	$1.0\times 10^{-5}$	4	0.707
#11	16	$1.0\times 10^{-5}$	2	0.655

**Table 3:** Model test performance on the ISIC 2017 dataset.

Model Name	Accuracy	Sensitivity	Specificity	AUROC
IRv2 + soft attention [33]	0.904	0.916	0.833	**0.959**
ARL-CNN50 [34]	0.868	0.878	0.867	0.958
SEnet50 [35]	0.863	0.856	0.865	0.952
RAN50 [36]	0.862	0.878	0.859	0.942
ResNet50 [37]	0.842	0.867	0.837	0.948
**Proposed ViT-based approach**	**0.948**	**0.928**	**0.967**	0.948

## Data Availability

ISIC Archive website https://challenge.isic-archive.com/data/#2017 (accessed on 1 June 2023).

## Abbreviations

The following abbreviations are used in this manuscript:

AI	Artificial intelligence
AUROC	Area under the receiver operating characteristic
CAD	Computer-aided diagnosis
CNNs	Convolutional neural networks
DL	Deep learning
e-Health	Electronic Health
FTNs	Fully Transformer networks
GANs	Generative adversarial networks
GELU	Gaussian error linear unit
IRNNs	Identity recurrent neural networks
ISIC	International Skin Imaging Collaboration
LSTM	Long short-term memory
m-Health	Mobile Health
ML	Machine learning
MM	Malignant melanoma
MLP	Multilayer perceptron
NCN	Nevocellular nevus
RCNNs	Recurrent convolutional neural networks
RNNs	Recurrent neural networks
SK	Seborrheic keratosis
SPTs	Spatial pyramid transformers
UV	Ultraviolet
ViTs	Vision transformers
WHO	World Health Organization

## References

[1] Hu W., Fang L., Ni R., Zhang H., Pan G. (2022). Changing trends in the disease burden of non-melanoma skin cancer globally from 1990 to 2019 and its predicted level in 25 years. BMC Cancer.

[2] Lacey J.V., Devesa S.S., Brinton L.A. (2002). Recent trends in breast cancer incidence and mortality. Environ. Mol. Mutagen..

[3] Uong A., Zon L.I. (2010). Melanocytes in development and cancer. J. Cell. Physiol..

[4] Siegel R.L., Miller K.D., Goding Sauer A., Fedewa S.A., Butterly L.F., Anderson J.C., Cercek A., Smith R.A., Jemal A. (2020). Colorectal cancer statistics, 2020. CA Cancer J. Clin..

[5] Verma R., Anand S., Vaja C., Bade R., Shah A., Gaikwad K. (2016). Metastatic malignant melanoma: A case study. Int. J. Sci. Study.

[6] Naik P.P. (2021). Cutaneous malignant melanoma: A review of early diagnosis and management. World J. Oncol..

[7] Jutzi T.B., Krieghoff-Henning E.I., Holland-Letz T., Utikal J.S., Hauschild A., Schadendorf D., Sondermann W., Fröhling S., Hekler A., Schmitt M. (2020). Artificial intelligence in skin cancer diagnostics: The patients’ perspective. Front. Med..

[8] Pollastri F., Parreño M., Maroñas J., Bolelli F., Paredes R., Ramos D., Grana C. (2021). A deep analysis on high-resolution dermoscopic image classification. IET Comput. Vis..

[9] Lucieri A., Bajwa M.N., Braun S.A., Malik M.I., Dengel A., Ahmed S. (2022). ExAID: A multimodal explanation framework for computer-aided diagnosis of skin lesions. Comput. Methods Programs Biomed..

[10] Pollastri F., Bolelli F., Paredes R., Grana C. (2020). Augmenting data with GANs to segment melanoma skin lesions. Multimed. Tools Appl..

[11] Aljohani K., Turki T. (2022). Automatic Classification of Melanoma Skin Cancer with Deep Convolutional Neural Networks. Ai.

[12] Allugunti V.R. (2022). A machine learning model for skin disease classification using convolution neural network. Int. J. Comput. Program. Database Manag..

[13] Malo D.C., Rahman M.M., Mahbub J., Khan M.M. Skin Cancer Detection using Convolutional Neural Network. Proceedings of the 2022 IEEE 12th Annual Computing and Communication Workshop and Conference (CCWC).

[14] Yamashita R., Nishio M., Do R.K.G., Togashi K. (2018). Convolutional neural networks: An overview and application in radiology. Insights Imaging.

[15] Liang M., Hu X. Recurrent convolutional neural network for object recognition. Proceedings of the IEEE Conference on Computer Vision and Pattern Recognition.

[16] Chandra B., Sharma R.K. On improving recurrent neural network for image classification. Proceedings of the 2017 International Joint Conference on Neural Networks (IJCNN).

[17] Dosovitskiy A., Beyer L., Kolesnikov A., Weissenborn D., Zhai X., Unterthiner T., Dehghani M., Minderer M., Heigold G., Gelly S. (2021). An Image is Worth 16x16 Words: Transformers for Image Recognition at Scale. arXiv.

[18] Bhojanapalli S., Chakrabarti A., Glasner D., Li D., Unterthiner T., Veit A. Understanding robustness of transformers for image classification. Proceedings of the IEEE/CVF International Conference on Computer Vision.

[19] Lanchantin J., Wang T., Ordonez V., Qi Y. General multi-label image classification with transformers. Proceedings of the IEEE/CVF Conference on Computer Vision and Pattern Recognition.

[20] Xie J., Wu Z., Zhu R., Zhu H. Melanoma detection based on swin transformer and SimAM. Proceedings of the 2021 IEEE 5th Information Technology， Networking, Electronic and Automation Control Conference (ITNEC).

[21] Roy V.K., Thakur V., Goyal N. (2023). Vision Transformer Framework Approach for Melanoma Skin Disease Identification. https://assets.researchsquare.com/files/rs-2536632/v1/00ee7438-9206-4cfd-a8ad-319813d22bb8.pdf?c=1682720069.

[22] Codella N.C.F., Gutman D., Celebi M.E., Helba B., Marchetti M.A., Dusza S.W., Kalloo A., Liopyris K., Mishra N., Kittler H. (2018). Skin Lesion Analysis Toward Melanoma Detection: A Challenge at the 2017 International Symposium on Biomedical Imaging (ISBI), Hosted by the International Skin Imaging Collaboration (ISIC). arXiv.

[23] Cubuk E.D., Zoph B., Shlens J., Le Q.V. (2019). RandAugment: Practical automated data augmentation with a reduced search space. arXiv.

[24] Hendrycks D., Gimpel K. (2020). Gaussian Error Linear Units (GELUs). arXiv.

[25] Devlin J., Chang M.W., Lee K., Toutanova K. (2019). BERT: Pre-training of Deep Bidirectional Transformers for Language Understanding. arXiv.

[26] Florkowski C.M. (2008). Sensitivity, specificity, receiver-operating characteristic (ROC) curves and likelihood ratios: Communicating the performance of diagnostic tests. Clin. Biochem. Rev..

[27] Deng J., Dong W., Socher R., Li L.J., Li K., Fei-Fei L. ImageNet: A large-scale hierarchical image database. Proceedings of the 2009 IEEE Conference on Computer Vision and Pattern Recognition.

[28] Gulzar Y., Khan S.A. (2022). Skin Lesion Segmentation Based on Vision Transformers and Convolutional Neural Networks—A Comparative Study. Appl. Sci..

[29] He X., Tan E.L., Bi H., Zhang X., Zhao S., Lei B. (2022). Fully transformer network for skin lesion analysis. Med. Image Anal..

[30] Azad R., Asadi-Aghbolaghi M., Fathy M., Escalera S. (2020). Attention deeplabv3+: Multi-level context attention mechanism for skin lesion segmentation. Proceedings of the Computer Vision—ECCV 2020 Workshops.

[31] Wu W., Mehta S., Nofallah S., Knezevich S., May C.J., Chang O.H., Elmore J.G., Shapiro L.G. (2021). Scale-aware transformers for diagnosing melanocytic lesions. IEEE Access.

[32] Aladhadh S., Alsanea M., Aloraini M., Khan T., Habib S., Islam M. (2022). An effective skin cancer classification mechanism via medical vision transformer. Sensors.

[33] Datta S.K., Shaikh M.A., Srihari S.N., Gao M. (2021). Soft-Attention Improves Skin Cancer Classification Performance. medRxiv.

[34] Zhang B., Jin S., Xia Y., Huang Y., Xiong Z. (2019). Attention Mechanism Enhanced Kernel Prediction Networks for Denoising of Burst Images. arXiv.

[35] Hu J., Shen L., Sun G. Squeeze-and-excitation networks. Proceedings of the IEEE Conference on Computer Vision and Pattern Recognition.

[36] Wang F., Jiang M., Qian C., Yang S., Li C., Zhang H., Wang X., Tang X. (2017). Residual Attention Network for Image Classification. arXiv.

[37] He K., Zhang X., Ren S., Sun J. (2015). Deep Residual Learning for Image Recognition. arXiv.

